# Humidity-controlled heat treatment of fresh spinach noodles for color preservation and storage quality improvement

**DOI:** 10.1016/j.fochx.2023.101042

**Published:** 2023-12-03

**Authors:** Jun-jie Xing, Ling-ling Cheng, Shuai Feng, Xiao-na Guo, Ke-xue Zhu

**Affiliations:** State Key Laboratory of Food Science and Technology, School of Food Science and Technology, Jiangnan University, 1800 Lihu Avenue, Wuxi 214122, Jiangsu Province, PR China

**Keywords:** Hydrothermal treatment, Fresh spinach noodles, Color preservation, Polyphenol oxidase, Shelf life

## Abstract

The high sensitivity to color browning during room-temperature storage was a significant factor in limiting the development of fresh spinach noodles (FSN). The practice of humidity-controlled heat treatment (HCHT) at varying temperatures, relative humidity, and time was carried out to limit enzyme activity and improve the quality of FSN. Results showed that HCHT could maximize the color preservation of fresh spinach noodle quality while effectively inactivating polyphenol oxidase and the yeasts, and mold count in FSN during storage was almost undetectable after mild conditions (80 °C). The hardness and chewiness of HCHT noodles were significantly increased, but the free sulfhydryl content was reduced. At 80 °C, 90 %, 5 min, protein structural aggregation was found in the microstructure of HCHT fresh spinach noodles. HCHT also caused partial gelatinization, as evidenced by the decrease in starch gelatinization enthalpy from 5.49 to 4.77 J/g, although the gelatinization degree of FSN was comparatively low.

## Highlights

• Humidity-controlled heat treatment (HCHT) extends shelf life of fresh spinach noodles.
• HCHT protected the color protection of spinach noodles by inactivating PPO enzyme.
• Only 5 min of high temperature HCHT could eliminate yeast and mold in noodles.
• The starch gelatinization degree of HCHT fresh spinach noodle was relatively low.

## Graphical abstract

Humidity-controlled heat treatment (HCHT) was innovatively applied to improve the color preservation of fresh spinach noodles by inactivating PPO enzyme and reducing the chlorophyll degradation and carotene liberation.

**Figure:**
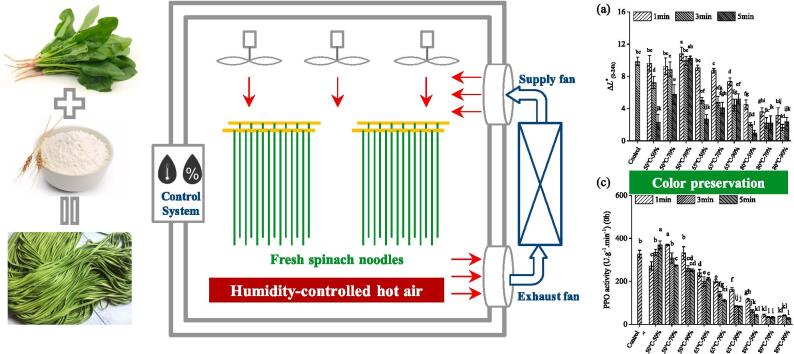

## 1 Introduction

Because spinach has anti-inflammatory, anti-proliferative, anti-obesity, and hypoglycemic physiological effects, spinach extract has been reported to be utilized as food fortification (Bunea et al., 2008, Shere et al., 2020). Fresh spinach noodles (FSN), which are nourishing, delectable, and appealing due to their vibrant green color, might be made by mixing raw spinach extract or spinach juice with wheat flour (Shere et al., 2020, Susanti et al., 2021). This product is very popular because it contains various vitamins and minerals that the human body needs, such as magnesium, potassium, iron vitamin K, vitamin A, folate, and so on. It occupies a large market share in fruit and vegetable noodles, especially during the COVID-19 pandemic. However, because of its high moisture and nutritional content, this type of fresh wet noodle (FWN) is prone to microbial proliferation during storage, resulting in a very short shelf life (Xing, Jiang, Yang, Guo, & Zhu, 2021). Recent years have seen advancements in the preservation of fresh wet noodles with the addition of organic acid, edible alcohol, and natural and synthetic preservatives as well as by lowering the water activity of the noodles (Ma, Sun, Li, & Zhu, 2020). Although the preservation technology has improved, the problem of color browning of spinach wet noodles still exists, and the storage process will lead to changes in appearance, which is not conducive to industrial production. Therefore, further research and exploration are needed to solve this problem in the future (Morris, 2018; Yang Zhao, Huang, Zhou, Zhang, & Ma, 2022).

According to Asenstorfer, Appelbee, and Mares (2009) and Zhao et al. (2023), non-enzymatic browning and chlorophyll degradation can result in discoloration of wheat flour-based FWN products. The main cause of this discoloration is a high activity of polyphenol oxidase (PPO). PPO catalyzes the conversion of monophenols into o-diphenols, which are then oxidized into o-quinones and water in the presence of oxygen (Y. Zhao et al., 2020). These o-quinones then interact with thiols, amines, and other functional groups of PPO, resulting in the creation of a dark material. This process leads to the browning of the noodles. Martin *et al.* reported that fresh raw noodles with lower PPO activity would have larger L* values and brown more slowly than noodles with higher PPO activity (Martin et al., 2005). To address this, methods should be explored to reduce PPO activity during the production process, thus improving the storage stability of FSN.

The color browning of FWN might be successfully delayed by chemical or enzymatic methods (Yang et al., 2014). Browning inhibition of whole wheat bread was observed to be efficient when ascorbic acid was coupled with cysteine (Zhao et al., 2020). Xylanase and papain were found to inhibit whole wheat dough browning by Yang et al. (2014) by acting on the phenolic compounds and polyphenol oxidase. Meanwhile, dry heat treatment with physical high and moderate temperatures and heat-moisture treatment may be considered green and advantageous strategies for color protection by reducing PPO activity (Wang et al., 2020, Yu et al., 2019). Zhao et al. (2023) heated wheat flour at 120 ℃ for 6 h and found that 95 percent of the PPO activity was lost. Yadav, Patki, Sharma, and Bawa (2008) studied the effect of microwave heating on the PPO activity of wheat grains with different moisture contents, They found that the PPO activity increased significantly when the moisture content of wheat seeds increased. High-humidity hot air impingement blanching with varied relative humidity, blanching temperature, and time was additionally employed for lowering the initial microbial load and inactivating peroxidase enzymes (POD) (Wang et al., 2020). In our previous study, we found that humidity-controlled hot air could be used to reduce microbial growth and improve the quality of fresh wet noodles (Xing et al., 2021). It is hypothesized that the fresh spinach noodles would benefit a lot from this environmentally friendly heat treatment with moderate or mild conditions, but the impact remained unknown. In this study, the effects of three independent humidity-controlled heat treatment (HCHT) variables including moderate temperature (65–80 °C), relative humidity (50 %–90 %), and time (1–5 min) on the color protection of FSN will be determined, and we will also investigate their influence on the storage quality and textural characteristics.

## 2 Materials and methods

### 2.1 Materials

Wheat flour was provided by Yihai Kerry Food Co., Ltd., China. Spinach was obtained from China Resources Vanguard supermarket. Catechol was obtained from Shanghai Aladdin Biochemical Technology Co., Ltd. Methanol, acetone, and phosphate buffer salt were provided by Sinopharm Chemical Reagent Co., Ltd and were of analytical grade.

### 2.2 Preparation of fresh spinach noodles (FSN)

Fresh spinach was carefully selected, washed, and drained thoroughly. Then add spinach and ice water (4 °C) in a ratio of 2:3 to the blender for juice extraction. Filter the spinach juice with eight layers of gauze and then store it below 4 °C in darkness before use. The fresh spinach noodles formula consisted of 400 g of flour and 124 g of spinach juice. The crumbly dough was prepared using a miniature mixer (Model HWJZ-5, Nanjing Yangzi Grain and Oil Food Processing Machinery Co., Ltd., Nanjing, China) and mixed for 5 min. Then sealed in a plastic bag resting for 20 min at 25 °C, and passed through the roller unit of an experimental noodle machine (Model JMTD-168/140, Dongfang Fude Technology Development Center, Beijing, China) with a roller gap to prepare noodles with dimensions of 2.0 mm in width and 1.0 mm in thickness.

### 2.4 Humidity-controlled heat treatment (HCHT)

The fresh spinach noodles were subjected to humidity-controlled heat treatment with automatic experimental equipment (Model SYT-030, China Packaging and Food Machinery Co., Ltd., Beijing, China). The noodles were treated under different treatment temperatures (50, 65, and 80 °C), relative humidity (50 %, 70 %, and 90 %), and treatment time (1, 3, and 5 min). Before packaging, HCHT noodle samples were cooled for 1 h under ambient conditions at around 25 °C to ensure uniform moisture distribution, and the FSN without HCHT was packaged as the control group. The water content for the control sample and HCHT noodle samples were listed in S-Table 1. Part of the noodle samples was lyophilized, ground, and passed through 80 mesh before analysis.

### 2.5 Color measurement of HCHT noodles

Color measurement of HCHT and fresh spinach noodle samples was performed by a Minolta chromameter (Model CR-400, Minolta Camera Co., Osaka, Japan). The color changes were expressed as L* (L*, black to white, 0 to 100, respectively), a* (ranging from red to green, positive to negative scale, respectively), and b* (ranging from yellow to blue, positive to negative scale, respectively). Three testing points were randomly selected and averaged on the front and background of the fresh spinach noodles. Color change (ΔL*_(0-24h)_, Δa*_(0-24h)_, and Δb*_(0-24h)_) was also obtained by measuring the color after 24 h storage, and total color change ΔE*_(0-24h)_ was calculated by the following equation.

$$\Delta E^{*}=\sqrt{{\left(\Delta L^{*}\right)}^{2}+{\left(\Delta a^{*}\right)}^{2}+{\left(\Delta b^{*}\right)}^{2}}$$

### 2.6 Determination of polyphenol oxidase (PPO) activities

The method of PPO extraction was carried out as described by Yadav et al. (2008) with slight modifications. The noodle sample powders (4 g) were mixed with phosphate-buffered saline (10 mL, 0.1 M, pH 6.0) and stirred for 48 h at 4 °C. The suspension was centrifuged at 10615 × g for 20 min at 4 °C. The supernatant was obtained as crude PPO extract and kept at 4 °C. The stored crude extract (250 μL) was added into a reaction mixture consisting of 50 μL of sodium phosphate buffer (0.1 mol/L, pH 6.0) containing 0.1 mmol/L catechol solution. The PPO activity was determined by measuring the increased absorbance at 420 nm with a preheated Epoch 2 microplate spectrophotometer (Model EPOCH2T, BioTek In-struments Inc., Winooski, Vermont, USA).

### 2.7 Determination of chlorophyll content

Chlorophyll content was assayed as described by Weemaes, Ooms, Loey, and Hendrickx (1999)_ENREF_33 with some modifications. The noodle sample powders (2.5 g) were mixed with 10 mL 80 % acetone solution and then homogenized for 10 min. The sample was centrifuged at 10000 × g for 5 min at 4 °C. The extraction procedure was repeated four times. The supernatant was filtered and then the absorbance of the extract was measured using an ultraviolet spectrophotometer (Model TU-1810, PERSIE, Beijing, China) at 663, and 645 nm. The calculation is as follows:

$$Totalchlorophyll\left(\mathrm{mg}/L\right)=20.21D_{645}+8.02D_{663}$$

$$Chlorophylla\left(\mathrm{mg}/L\right)=12.7D_{663}-2.69D_{645}$$

$$Chlorophyllb\left(\mathrm{mg}/L\right)=22.9D_{645}-4.68D_{663}$$

### 2.8 Determination of free phenolic content

The free phenolic content of HCHT and fresh spinach noodle samples was measured as described by Levent (2017) with some modifications. The standard curve was first determined by using gallic acid and quantified colorimetrically at 750 nm. Noodle sample powders (0.2 g) were extracted with 70 % methanol (4 mL) preheated at 70 °C for 30 min and then homogenized for 10 min. After that, the samples were centrifuged at 1258 × g for 5 min at 4 °C. The supernatants were collected and diluted to 10 mL with 70 % methanol. The supernatant (1 mL) was then mixed with Folin-Ciocalteu reagent (4 mL, 10 % v/v). Three minutes later, 5 mL Na_2_CO_3_ solution (7.5 % v/v) was added. The mixture was then kept in the dark at ambient temperature for 1 h. The absorbance of the mixture was measured at 765 nm using the spectrophotometer to determine the free phenolic content.

### 2.9 Determination of carotenoid content

The carotenoid content of HCHT and fresh spinach noodles was determined according to the AACC method 14–50 (AACC, 2000a) by mixing lyophilized spinach noodle powder (2 g) with 10 mL of water-saturated *n*-butanol solution and shaking the extraction for 1 h. The supernatant was centrifuged (25 °C, 10,000 r/min, 10 min) after standing at room temperature for 15 min to extract the supernatant, which was filtered and the absorbance of the extract was measured at 440 nm.

### 2.10 Microbial content analysis

The HCHT and fresh spinach noodles (25 g) in different storage stages were sampled, pulverized, and mixed with 225 mL of 0.85 % aseptic physiological saline. The mixture was homogenized by a stomacher machine (Lab-blender 400; Seward Laboratory) for 60 s before being transferred to make different series of dilutions using 0.85 % aseptic physiological saline. 1 mL of appropriate dilutions was pipetted onto sterile plate count agar plates and incubated at 36 °C for 48 h before the examination of the total plate counts (TPC). The yeasts and molds count (YMC) were also calculated using Bengal red medium after incubation for 5 days at 28 °C.

### 2.11 Noodle quality characteristics

The texture properties of HCHT and fresh spinach noodles were analyzed by TA-XT2i Texture Analyzer (Stable Micro Systems, London, England). The cooked noodles were drained under cold water. Two 10 cm intact noodles were placed on the carrier table for full mass structure analysis. The probe model was P36R with a pre-test speed of 2 mm/s and a post-test and post-test speed of 0.8 mm/s. The compression deformation ratio was 75 % and the trigger force was 5 g. The noodle tensile strength was measured according to Xing et al. (2021) with slight modifications. The A/SPR type probe was selected and the ends of the noodles were wrapped around the upper and lower arms of the probe. The starting distance was 10 mm, the pre-test speed was 2 mm/s, the test speed was 3 mm/s, the post-test speed was 10 mm/s, the pull-off distance was 150 mm, and the trigger force was 5 g.

In addition, the cooking loss of FSN was determined according to AACC 66–50 (AACC, 2000b) with slight modifications. Fresh spinach noodles were cooked in boiling water for the optimal cooking time and then retrieved. The cooking water was cooled to room temperature and transferred to a 500 mL volumetric flask for volume determination, and then 100 mL of it was transferred to a 250 mL beaker and boiled to evaporate the water. When the water in the beaker evaporates to about 5 mL, place it into an air oven at 105 °C until a constant weight is reached. The cooking loss is calculated as follows.

$$Cooking\;loss\;\left(\%\right)=\frac{5\times M}{m\times \left(1-w\right)}$$

where M (g) is the weight of dry matter in the cooling water, m (g) is the weight, and w (%) is the water content of fresh noodles.

### 2.12 Pasting properties

The pasting properties of HCHT and fresh spinach noodles were determined by a rapid viscosity analyzer (RVA 4500, Perten Instruments Australia). The noodle sample powders (3.5 g) were mixed with 25 mL of deionized water in an aluminum box. The temperature variation was set to equilibrate at 50 °C for 1 min, heated to 95 °C at 12 °C/min, and maintained at this temperature for 3.5 min. Finally, sample pastes were cooled down to 50 °C at 12 °C/min and held for 2 min.

### 2.13 Thermal properties

The thermal properties of HCHT and fresh spinach noodles were examined by a differential scanning calorimeter (DSC3, Mettler-Toledo, Switzerland). The spinach noodle powder (2 mg) with 6 mg of deionized water was accurately weighed in a 40 µL aluminum pan. The aluminum trays were sealed and equilibrated at 4 °C for 12 h. The sample was heated from 25 °C to 100 °C at a rate of 10 °C/min. The onset, peak, and conclusion temperature (T_o_, T_p_, and T_c_) together and enthalpy change (ΔH) were determined with the thermal analysis software.

### 2.14 Microstructure analysis

To analyze the microstructure, the HCHT and fresh spinach noodles were first freeze-embedded with Leica gel. After stereotyping, the samples were cut into 10 μm slices by a frozen sectioning machine and the slices were placed on slides. The slides were stained with a mixture of 0.25 % fluorescein isothiocyanate and 0.025 % rhodamine B for 1 min. The slides were then rinsed with deionized water and excess water was blotted out. A confocal laser scanning microscope (CLSN, Model LSM 710, Leica, Germany) was used to observe the microstructure of the noodles.

### 2.15 Statistical analysis

The data obtained in this study were expressed as the mean ± standard deviation (SD) with at least three replicate determinations by using the software SPSS 16.0. One-way analysis of variance (ANOVA) was used to statistically analyze the data. Significance was defined at *p* < 0.05 by using Duncan's test. All figures were plotted using Origin Pro 2018 (Origin Lab Corporation, Northampton, MA, USA).

## 3 Results and discussion

### 3.1 Color browning of HCHT fresh spinach nood1le

**Fig. 1:**
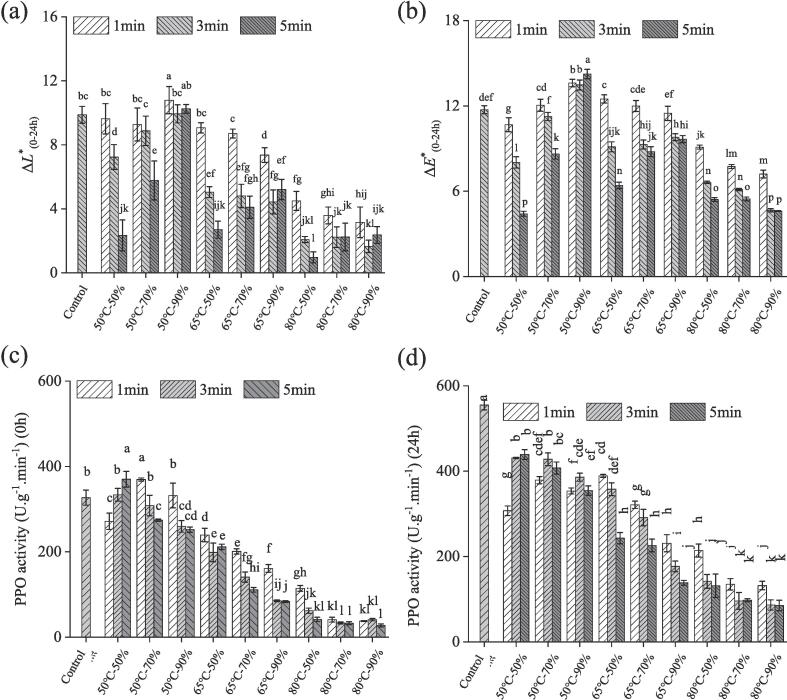
Effect of humidity-controlled heat treatment on the color changes and PPO activity of fresh spinach noodles over 24 h period.

This study aimed to investigate the influence of humidity-controlled heat treatment (HCHT) on the color change of fresh spinach noodles. By comparing the color difference of noodles before and after 24 h storage, the degree of color browning was measured. Results showed that the three independent variables of HCHT have different effects on the color change of noodles after 24 h of storage. In general, the ΔL*_(0-24h)_ decreased with the increase of HCHT time under the same temperature but longer HCHT time may not have a lower degree of browning. At the same time, the ΔL*_(0-24h)_ decreased with the increase of HCHT temperature under the same time, and the higher the humidity, the greater the decrease of ΔL*_(0-24h)_ (Fig. 1a). This is because more water was involved in the heat transfer process at higher RH conditions, resulting in faster inactivation of PPO in fresh noodles (Wang et al., 2020, Yu et al., 2019). As for the relative humidity (RH), the amount of water molecules involved in heat and mass transfer varied with temperature. The ΔL*_(0-24h)_ of HCHT noodles treated at 50 °C increased with increasing relative humidity (RH), while it showed a significant decreasing trend with increasing relative humidity when treated at 65 °C (from 9.06 to 7.35) or 80 °C (from 4.50 to 3.16) for 1 min. Enzymatic browning caused by PPO is the main reason for fresh noodles browning, and its activity is highly dependent on the water content and water activity of FSN (Ma et al., 2020). The increase of ΔL*_(0-24h)_ of fresh noodles with RH at a relatively low temperature (50 °C) may be due to the increase in the water content of noodles after HCHT (S-Table 1). Asenstorfer et al. (2009) also reported that the water content of fresh noodles could reduce the reflectance of the surface of noodles by affecting the protein molecules and structure.

In addition, ΔE*_(0-24h)_ is also a parameter used to evaluate the color difference between noodles stored for 24 h and the fresh wet noodles. The changes of ΔE_(0-24h)_ with the increase of humidity varied under different mild temperatures during the storage of HCHT fresh spinach noodles. At 50 °C, the value increases with increasing humidity, however, this variation decreases with humidity when temperature increases up to 65 °C and 80 °C (Fig. 1b). The lowest ΔE*_(0-24h)_ of HCHT fresh spinach noodles was obtained at treatment conditions of 80 °C-90 %-5 min. These results showed that the humidity-controlled heat treatment could significantly inhibit the browning of fresh spinach noodles.

### 3.2 PPO activity of HCHT fresh spinach noodle

PPO is a crucial enzyme in enzymatic browning, and its activity serves as an extremely important indicator for evaluating and predicting the browning of fresh noodle color. As shown in Fig. 1c, after humidity-controlled heat treatment, the PPO activity of FSN shows a downward trend. However, under relatively low temperatures and humidity (HCHT of 50 °C-50 %), PPO activity increases with longer treatment time. Compared with the control group’s 326.8 U g^−1^·min^−1^, the PPO activity of the HCHT sample of 50 °C-50 %-5 min is highest, reaching 394.5 U g^−1^·min^−1^. Furthermore, as temperature rises, the PPO activity significantly decreases with an increase in relative humidity (*p* < 0.05). The surface temperature of noodles elevates more rapidly at higher humidity (Yu et al., 2019). With the increase of temperature and humidity, the PPO activity was further reduced, with the lowest activity of 41.5 U·g^−1^ min^−1^ observed in the noodle sample treated at 80 °C-90 %-5 min.

The changing trend of PPO activity in HCHT noodles (Fig. 1d) over 24 h is consistent with that in Fig. 1 (c). After 24 h of storage, the PPO activity of all HCHT groups of noodles increased significantly, while the PPO activity in the 80 °C treatment group remained lower than 214 U g^−1^ min^−1^ (compared to 555 U g^−1^ min^−1^ in the control group). The increase in PPO activity in noodles during storage may be attributed to the reversible structural or conformational changes in the PPO enzyme (Liu et al., 2009). In this study, the humidity-controlled heat treatment at 80 °C showed the best effect in inhibiting the color browning of fresh spinach noodles by reducing PPO activity. Therefore, we will primarily discuss the impact of HCHT-80 °C treatment on the qualities of fresh spinach noodles in subsequent discussions.

### 3.3 Chlorophyll content in HCHT fresh spinach noodle

**Fig. 2:**
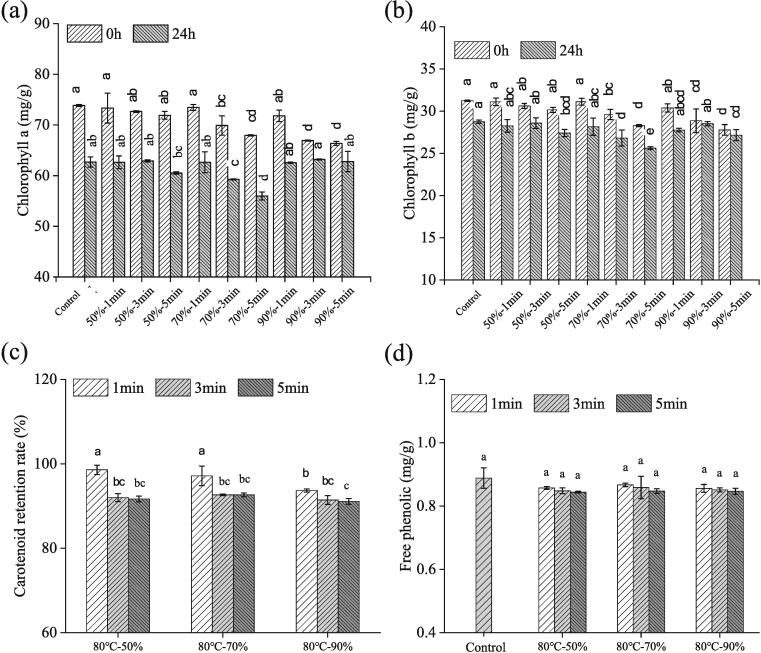
Effects of humidity-controlled heat treatment at 80 °C on chlorophyll, carotenoid retention, and free phenol content of fresh spinach noodles over 24 h period.

Chlorophyll is the main pigment of green plants and many studies have shown that chlorophyll has antimutagenic and anticarcinogenic activities (Simonich et al., 2008). Heat treatment has been reported to result in the replacement of Mg of chlorophyll by hydrogen and the conversion of chlorophyll to pheophytin, changing the color of the food from bright green to dark olive green or olive yellow (Yang Zhao et al., 2022). The effect of HCHT on chlorophyll *a* and b content in spinach fresh noodles is shown in Fig. 2 (a) and (b), which illustrate a clear trend of decreasing chlorophyll content with increasing HCHT time and relative humidity (RH). When the HCHT time was 1 min, there was no significant change in the chlorophyll content of spinach fresh noodles as RH increased. However, with the increase of RH from 70 % to 90 % and HCHT time from 3 to 5 min, a more pronounced decrease in chlorophyll content was observed. The degree of chlorophyll degradation was closely related to the intensity of heat treatment, which was determined by the treatment temperature, RH, and heating time (Benlloch-Tinoco et al., 2015). An increase in RH could improve the heat and mass transfer coefficient and increase the surface temperature of the samples, leading to the degradation of chlorophyll (Turkmen, Poyrazoglu, Sari, & Sedat Velioglu, 2006). Hence, HCHT with a relatively shorter time and lower RH generally had no significant effect on the chlorophyll content of FSN. These results highlight the importance of carefully controlling the HCHT conditions to minimize the degradation of chlorophyll in spinach fresh noodles.

After 24 h of storage, chlorophyll in all experimental groups degraded, but the rate of chlorophyll degradation on FSN was slowed down under the condition of 80 °C, 90 %, and 5 min. The decrease in chlorophyll *a* and chlorophyll *b* content during storage was attributed to the degradation of chlorophyll. Notably, when examining the effect of different humidity conditions on chlorophyll degradation, it was observed that the experimental group with 90 % relative humidity had the lowest total chlorophyll loss. Studies have shown that the degradation of chlorophyll is mainly caused by enzymatic degradation (deacetylmagnetase, chlorophyllase, chlorophyll oxidase, peroxidase, etc.) (Benlloch-Tinoco et al., 2015). Hydrothermal treatment not only reduced PPO activity but also inactivated enzymes related to chlorophyll degradation, thereby delaying the degradation of chlorophyll during noodle storage. Furthermore, the loss of chlorophyll *b* during storage was less than that of chlorophyll *a* in all FSN samples. This may be due to the greater stability of chlorophyll *b* compared to chlorophyll *a* during storage (Turkmen et al., 2006).

### 3.4 Carotenoids and free phenols in HCHT fresh spinach noodle

Spinach is a great source of carotenoids and its color is usually masked by the green chlorophyll (Bunea et al., 2008). The presence of carotenoids could lead to a reduction in the brightness (L*) of the FSN product. Fig. 2 (c) illustrates the effect of HCHT on the retention of carotenoids in FSN. Carotenoid retention showed a decreasing trend with increasing RH and HCHT time. It is worth mentioning that due to their unsaturated structural characteristics, carotenoids are prone to decomposition under light and heat conditions. Under the HCHT condition of the shortest time and lowest humidity (80 °C-50 %-1 min), the carotenoid retention of FSN was 98.6 %, while it decreased to 91 % under the contdition of 80 °C-90 %-5 min of HCHT. This indicated that the humidity-controlled hot air employed in this study had a less destructive effect on carotenoids in fresh spinach noodles. Chiremba, Pozniak, and Fu (2015) found that there was only a small loss of carotenoid in pasta after thermally drying at 85 °C for 325 min.

Phenolic compounds have many physiological functions such as antioxidant therapy for cardiovascular diseases (Bunea et al., 2008). Spinach is particularly rich in these phenolics, and when introduced into noodles, they elevate levels of free phenols in fresh spinach noodles. In this study, the control FSN sample had a free phenol content of approximately 0.88 mg/g, which is higher than reported in fresh white noodles. Interestingly, varying the relative humidity and time in HCHT had no significant effect on the free phenolic content in FSN (*p* > 0.05), as shown in Fig. 2 (d). Similarly, Chiremba et al. (2015) found no significant difference in the free and bound phenol content in pasta dried at 80 °C for 4 h compared to the undried group. Mazzeo et al. (2011) also showed that cooking spinach in boiling water for 10 min reduced its total phenolic content by only 4 %. Overall, the humidity-controlled heat treatment not only improves the color preservation of fresh spinach noodles by inactivating the PPO enzyme and reducing chlorophyll degradation and carotene liberation but also preserves the beneficial phenols in FSN to a great extent. In subsequent sections, we will explore the effects of HCHT at 80 °C on the storage qualities and textural properties of FSN.

### 3.5 Microbial content of HCHT fresh spinach noodles

**Fig. 3:**
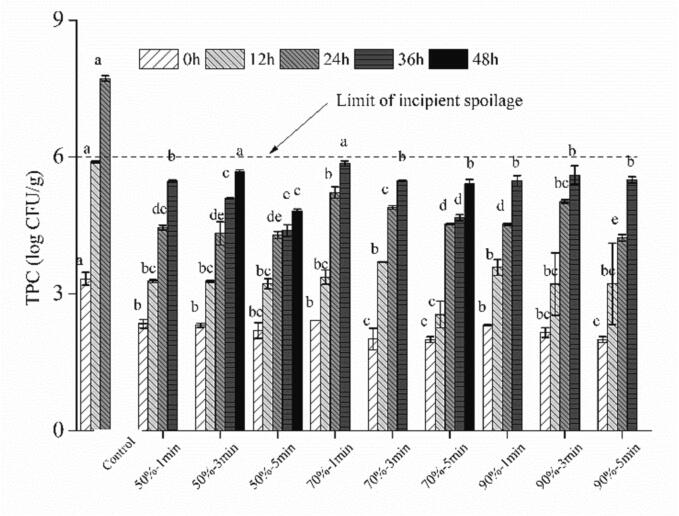
Effects of humidity-controlled heat treatment on total plate count of fresh spinach noodles during storage at 25 °C.

Generally, the shelf life of fresh spinach noodles in room-temperature storage is very short and is closely related to the initial microbial load. The TPC threshold level of 10^6^ CFU/g could be considered as the limit of incipient spoilage in fresh wet noodles, thus the quality analysis of fresh spinach noodle samples would be terminated in this study (Li et al., 2016). The initial TPC of fresh spinach noodles was 3.33 log_10_ CFU/g, exceeding the threshold level within 12 h of storage at 25 °C. In this study, humidity-controlled heat treatment at RH of 50 % for only 1 min would significantly reduce the initial TPC to 2.34 log_10_ CFU/g, but longer treatment time did not further decrease the TPC (Fig. 3). The initial TPC of HCHT samples was further decreased when the HCHT time increased to 5 min under higher RH (70 % and 90 %). When the HCHT was conducted at 80 °C, 90 % RH for 5 min, the noodle sample had the lowest initial TPC of 1.99 log_10_ CFU/g. As previously reported, increasing temperature was more effective in reducing bacteria compared to adjusting RH and time (Xing et al., 2021). The yeast and molds count of fresh spinach noodles was also greatly reduced to undetectable levels after HCHT (S-Table 2). It was challenging to find a balance between reducing the initial bacterial content of fresh noodles and improving the quality of fresh noodles. Since the color changes and PPO activity were sensitive to hydrothermal treatment, we have reduced the HCHT time to 1–5 min when compared with up to 18 min of humidity-controlled dehydration treatment in the last paper (Xing et al., 2021). This condition (80 °C, 50 % RH, and 5 min) proved to be optimal as it not only reduced the initial TPC significantly but also ensured that the TPC did not exceed the threshold level even after 48 h of storage. When combined with the analysis of color and PPO activity changes under the same HCHT conditions, we believed that the moderate temperature of heat moisture treatment was very helpful in promoting color protection and was efficient in killing yeasts and molds in the noodles. Thus, it brought benefits for extending the shelf life of fresh spinach noodles (Li et al., 2016).

### 3.6 Quality characteristics of HCHT fresh spinach noodles

The effect of humidity-controlled heat treatment on the texture and cooking quality of fresh spinach noodles is listed in Table 1. Under optimal cooking conditions, the textural results showed that the hardness and chewiness of cooked HCHT noodles increased significantly after humidity-controlled heat treatment. The tensile force and tensile distance of raw HCHT noodles increased significantly at RH of 50 % and 70 %, which may be attributed to the aggregation of the gluten network during the HCHT process. There was little difference, however, in tensile force and tensile distance for cooked HCHT noodles. This could be because the cooking process overridden the effects imposed by HCHT on the noodles (Xing et al., 2021). Thus, HCHT increased the ability of fresh noodles to resist breaking without affecting the quality of cooked noodles. Furthermore, the cooking loss of fresh spinach noodles was slightly reduced, possibly due to the gluten aggregation network enclosing starch granules and limiting starch swelling and molecule leaching (Li et al., 2016). The underlying reasons for these effects will be further discussed in terms of the thermal properties and microstructure of FSN in the following sections. Overall, in addition to its bright green appearance and long shelf-life, the humidity-controlled heat-treated FSN exhibited good textural and sensory qualities comparable to fresh noodles in this study.

**Table 1:** Effect of hydrothermal treatment (HCHT) at 80 °C with different humidity and treatment time on the quality of fresh spinach noodles.

Treatment		Fresh noodles					Cooked noodles			
RH	Time	Tensile force (g)	Tensile distance (mm)	Cooking loss (%)	Free sulfhydryl content (μmol/g)		Tensile force (g)	Tensile distance (mm)	Hardness (g)	Chewiness (g)
Control		18.32 ± 1.38^f^	9.52 ± 1.32^ef^	6.97 ± 0.16^a^	3.65 ± 0.10^a^		22.07 ± 0.55^ab^	76.36 ± 2.74^abc^	1602.9 ± 27.3^d^	1238.3 ± 29.6^a^
50 %	1 min	23.96 ± 0.94^e^	21.33 ± 2.61^a^	6.73 ± 0.08^abcd^	2.68 ± 0.24^b^		22.22 ± 1.21^ab^	72.26 ± 1.86^abc^	1843.2 ± 48.3^b^	1430.5 ± 35.4^b^
	3 min	31.55 ± 1.37^c^	13.12 ± 3.35^de^	6.70 ± 0.13^abcd^	2.43 ± 0.42^bc^		22.71 ± 0.28^a^	71.25 ± 6.16^abc^	1879.4 ± 42.9^ab^	1489.8 ± 49.4^b^
	5 min	40.51 ± 2.27^a^	14.19 ± 5.86^cd^	6.60 ± 0.12^cd^	2.17 ± 0.18^cd^		22.40 ± 0.4^ab^	73.09 ± 5.67^bc^	1959.4 ± 17.5^a^	1477.9 ± 23.5^b^
70 %	1 min	24.13 ± 1.45^e^	18.80 ± 3.48^ab^	6.91 ± 0.08^abc^	2.04 ± 0.08^cde^		21.93 ± 1.27^ab^	67.56 ± 8.06^abc^	1884.8 ± 18.8^ab^	1416.2 ± 72.1^b^
	3 min	29.07 ± 1.34^d^	16.84 ± 2.20^bc^	6.91 ± 0.11^ab^	1.99 ± 0.18^de^		22.95 ± 0.13^a^	74.34 ± 3.97^abc^	1880.4 ± 66.5^ab^	1430.8 ± 58.5^b^
	5 min	36.92 ± 1.94^b^	10.88 ± 0.66^de^	6.60 ± 0.01^bcd^	2.09 ± 0.24^bcd^		20.95 ± 0.10^b^	67.10 ± 2.84^a^	1916.4 ± 66.3^ab^	1434.8 ± 16.1^b^
90 %	1 min	17.55 ± 1.14^f^	5.56 ± 0.53^g^	6.71 ± 0.09^abcd^	2.10 ± 0.15^bcd^		20.94 ± 0.43^b^	67.73 ± 1.87^bc^	1744.3 ± 65.7^c^	1401.1 ± 45.8^b^
	3 min	19.56 ± 1.67^f^	5.55 ± 1.08 ^g^	6.75 ± 0.12^abcd^	2.03 ± 0.11^cde^		21.06 ± 0.47^b^	62.95 ± 4.38^a^	1719.1 ± 52.0^c^	1401.7 ± 93.5^b^
	5 min	19.41 ± 1.48^f^	6.08 ± 1.33 ^fg^	6.48 ± 0.21^d^	1.65 ± 0.23^e^		21.79 ± 0.39^ab^	66.17 ± 1.19^ab^	1743.8 ± 56.4^c^	1413.1 ± 125.4^b^

Values of moisture content represent mean ± S.D., n = 3. The different lowercase letter means significant difference within this table (*P* < 0.05).

### 3.7 Thermal properties of HCHT fresh spinach noodle

The humidity-controlled heat treatment affected the starch component in FSN by energy input. The water molecule in hot air, as the medium, could affect the heat transfer efficiency (Dutta & Mahanta, 2012). The pasting and thermal properties of fresh spinach noodles after humidity-controlled heat treatment are listed in Table 2. Results showed that the peak viscosity and breakdown viscosity decreased with increasing relative humidity, while the final viscosity of noodle starch increased significantly. Hormdok and Noomhorm reported a decrease in the peak viscosity of rice starch after 1.5 h of humidity-controlled heat treatment at 110 °C (Hormdok & Noomhorm, 2007). Luo *et al*., also investigated the effects of cooking on quick-frozen noodles, finding similar decreases in breakdown values (Luo, Guo, & Zhu, 2015). The thermal properties revealed significant increases in onset and peak temperatures with rising RH. Additionally, Luo et al. (2015) found that heat moisture treatment enhanced crystalline structures, requiring more energy to disrupt. While there was no significant difference in enthalpy between 50 % and 70 % RH treatments compared to controls, enthalpy decreased from 5.49 J/g to 4.77 J/g at 90 % RH. This decrease may be attributed to increased water molecule involvement in starch gelatinization. Notably, the loss ratio of starch gelatinization enthalpy in HCHT noodles at 80 °C, 90 %, and 5 min was only about 13 %, indicating that all HCHT noodle groups could be considered as raw fresh noodles.

**Table 2:** Effect of humidity-controlled heat treatment (HCHT) on pasting and thermal properties of fresh spinach noodles.

Treatment	RVA characteristics					DSC characteristics			
	Gelatinization temperature	Peak viscosity (cP)	Final viscosity (cP)	Breakdown viscosity (cP)		To (^o^C)	Tp (^o^C)	Tc (^o^C)	ΔH (J g^−1^)
Control	70.45 ± 1.13^a^	2422.50 ± 6.36^a^	2478.0 ± 67.88^c^	931.5 ± 38.89^a^		56.41 ± 0.08^d^	63.20 ± 0.06^d^	68.94 ± 0.04^c^	5.49 ± 0.37^ab^
80℃-50 %-5 min	68.43 ± 0.60^b^	2494.00 ± 15.56^a^	3175.5 ± 4.24^a^	635 ± 10.61^b^		60.70 ± 0.17^c^	64.73 ± 0.13^c^	68.96 ± 0.10^c^	6.07 ± 0.13^a^
80℃-70 %-5 min	69.23 ± 0.67^a^	2212.00 ± 14.14^b^	2999.0 ± 29.7^b^	491.0 ± 33.94^c^		62.80 ± 0.01^b^	66.06 ± 0.11^b^	69.97 ± 0.15^b^	5.34 ± 0.21^bc^
80℃-90 %-5 min	70.08 ± 0.60^a^	1978.50 ± 4.95^c^	2907.0 ± 36.77^b^	334.0 ± 15.56^d^		64.00 ± 0.23^a^	67.23 ± 0.13^a^	70.77 ± 0.11^a^	4.77 ± 0.03^c^

Values of moisture content represent mean ± S.D., n = 3. The different lowercase letter means significant difference within this table (*P* < 0.05).

### 3.8 Microstructure of HCHT fresh spinach noodle

**Fig. 4:**
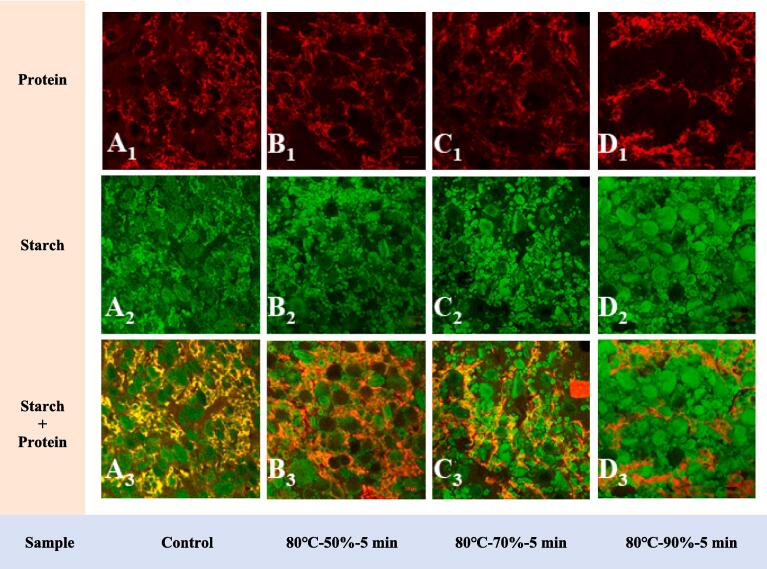
Effect of humidity-controlled heat treatment on the microstructure of fresh spinach noodles. Note: A_1_-A_3_: Control group protein, starch, and composite structure; B_1_-B_3_: protein, starch, and composite structure of fresh spinach noodles after 80 °C, 50 % relative humidity for 5 min treatment; C_1_-C_3_: protein, starch, and composite structure of fresh spinach noodles after 80 °C, 70 % relative humidity for 5 min treatment; D_1_-D_3_: protein, starch and composite structure of fresh spinach noodles after 80 °C, 90 % relative humidity for 5 min treatment.

In this paper, HCHT noodles treated at 80 °C for 5 min with different relative humidity exhibited relatively higher strength. Thus, the microstructure of these noodles could reflect the dramatic changes in their external and internal properties after HCHT. As RH increased, the HCHT conditions did not significantly alter the microstructure of the noodles at RH of 50 % and 70 %. However, the protein networks of the fresh spinach noodles aggregated as RH increased to 90 % (Fig. 4A1-D1). The changes in the free sulfhydryl content of HCHT noodles were also consistent with the microstructure changes (Table 1). Under HCHT conditions of 80 °C-90 %-5 mintures, the free sulfhydryl content decreased to 1.65 μmol/g from 3.65 μmol/g of the control sample. When the temperature of the wheat dough is higher than 60 °C, hydrophobic groups like free sulfhydryl in wheat gluten were exposed to oxidization, resulting in the formation of disulfide bonds in or between molecules and protein polymerization begins inside the wheat dough (K. Q. Wang et al., 2017). Concerning starch granules in noodles, HCHT at 50 % and 70 % RH did not result in significant changes in the fresh spinach noodles and it still presented a typical form of raw wheat starches (Fig. 4A2-D2). One could tell that the starch granules in HCHT noodles at a higher RH of 90 % tend to swell and increase in volume, but no obvious starch paste was observed, which is consistent with the low starch gelatinization degree mentioned earlier. In general, the humidity-controlled heat treatment influenced the noodle structure in terms of starch gelatinization and protein aggregation, which in turn affected or promoted the textural properties of HCHT noodles. Therefore, this physical technique of HCHT was overall acceptable in terms of its influence on the quality of noodles, considering the benefits of color protection and shelf-life extension that it provides.

## 4 Conclusions

The humidity-controlled heat treatment has been innovatively applied to control microbial growth in fresh wet noodles. This study further focused on the effect of HCHT on the green color protection and quality improvement of fresh spinach noodles. HCHT with higher temperatures contributed to the color protection of spinach noodles by inactivating the PPO enzyme. The increase of relative humidity at higher temperature could greatly increase the heat transfer efficiency and less than 5 min of HCHT were effective in killing yeast and mold in noodles, thus extending the shelf-life of fresh spinach noodles. In addition, the retention rate of bioactive components in noodles like chlorophyll, carotenoids, and free phenols was still relatively high. The effect of HCHT on the structure and properties of protein and starch improved the textural quality of FSN to some extent. However, relying solely on physical treatment methods is insufficient to achieve the goal of color preservation. In the future, it is necessary to continue exploring comprehensive preservation methods, based on ensuring storage quality, to extend the shelf life of fresh spinach noodles.

## CRediT authorship contribution statement

**Jun-jie Xing:** Funding acquisition, Investigation, Validation, Methodology, Project administration, Writing – original draft, Writing – review & editing. **Ling-ling Cheng:** Data curation, Formal analysis, Methodology, Software, Writing – review & editing. **Shuai Feng:** Methodology, Validation, Formal analysis. **Xiao-na Guo:** Conceptualization, Investigation, Supervision, Validation. **Ke-xue Zhu:** Conceptualization, Investigation, Supervision, Visualization.

## Declaration of competing interest

The authors declare that they have no known competing financial interests or personal relationships that could have appeared to influence the work reported in this paper.

## Data Availability

Data will be made available on request.
