# Supplementary material for: Humidity-controlled heat treatment of fresh spinach noodles for color preservation and storage quality improvement
Source: Food Chem X. 2023 Dec 3;20:101042. doi: 10.1016/j.fochx.2023.101042 (PMC10740017; doi:10.1016/j.fochx.2023.101042)
Supplement: Supplementary data 1 [file mmc1.docx]

#### **S-Table 1.** Effects of humidity-controlled heat treatment (HCHT) on the water content of fresh spinach noodles

| Treatment^*^ | | Water content | | |
| --- | --- | --- | --- | --- |
|  |  | 1 min | 3 min | 5 min |
| Control | | 32.58±0.02^d^ | | |
| 50^o^C | 50% | 31.26±0.07^i^ | 29.86±0.03^ml^ | 29.18±0.03^o^ |
|  | 70% | 32.14±0.05^f^ | 30.88±0.07^k^ | 29.82±0.05^m^ |
|  | 90% | 32.85±0.01^c^ | 32.39±0.02^e^ | 31.91±0.03^h^ |
| 65^o^C | 50% | 32.06±0.04^gf^ | 29.82±0.06^m^ | 28.45±0.02^q^ |
|  | 70% | 32.87±0.11^c^ | 31.05±0.02^j^ | 30.01±0.22^l^ |
|  | 90% | 33.57±0.03^b^ | 32.68±0.06^d^ | 32.90±0.05^c^ |
| 80^o^C | 50% | 32.35±0.02^e^ | 29.60±0.05^n^ | 27.52±0.03^r^ |
|  | 70% | 32.19±0.05^f^ | 31.25±0.01^i^ | 28.62±0.01^p^ |
|  | 90% | 33.73±0.02^a^ | 32.54±0.01^d^ | 31.95±0.01^hg^ |

Values of moisture content represent mean±S.D., n = 3. Different lowercase letter means significant difference within this table (*P*<0.05).

#### **S-Table 2**. Effect of humidity-controlled heat treatment (HCHT) at 80 ^o^C on the yeasts and molds count of fresh spinach noodles (log_10_ CFU/g)

| Storage time (h) | Control | Treatment (min) | | | | | | | | |
| --- | --- | --- | --- | --- | --- | --- | --- | --- | --- | --- |
|  |  | 50% | | | 70% | | | 90% | | |
|  |  | 1 | 3 | 5 | 1 | 3 | 5 | 1 | 3 | 5 |
| 0 | 2.58±0.06 | <1 | <1 | <1 | <1 | <1 | <1 | <1 | <1 | <1 |
| 12 | 4.88±0.02 | <1 | <1 | <1 | <1 | <1 | <1 | <1 | <1 | <1 |
| 24 | — | <1 | <1 | <1 | <1 | <1 | <1 | <1 | <1 | <1 |
| 36 | — | <1 | <1 | <1 | <1 | <1 | <1 | <1 | <1 | <1 |

Values of moisture content represent mean±S.D., n = 3. Different lowercase letter means significant difference within this table (*P*<0.05).
